# Smart Construction of Integrated CNTs/Li_4_Ti_5_O_12_ Core/Shell Arrays with Superior High‐Rate Performance for Application in Lithium‐Ion Batteries

**DOI:** 10.1002/advs.201700786

**Published:** 2018-01-03

**Authors:** Zhujun Yao, Xinhui Xia, Cheng‐ao Zhou, Yu Zhong, Yadong Wang, Shengjue Deng, Weiqi Wang, Xiuli Wang, Jiangping Tu

**Affiliations:** ^1^ State Key Laboratory of Silicon Materials Key Laboratory of Advanced Materials and Applications for Batteries of Zhejiang Province School of Materials Science & Engineering Zhejiang University Hangzhou 310027 China; ^2^ School of Engineering Nanyang Polytechnic Singapore 569830 Singapore

**Keywords:** carbon nanotubes, conductive networks, lithium ion batteries, lithium titanate, ultrafast energy storage

## Abstract

Exploring advanced high‐rate anodes is of great importance for the development of next‐generation high‐power lithium‐ion batteries (LIBs). Here, novel carbon nanotubes (CNTs)/Li_4_Ti_5_O_12_ (LTO) core/shell arrays on carbon cloth (CC) as integrated high‐quality anode are constructed via a facile combined chemical vapor deposition–atomic layer deposition (ALD) method. ALD‐synthesized LTO is strongly anchored on the CNTs' skeleton forming core/shell structures with diameters of 70–80 nm the combined advantages including highly conductive network, large surface area, and strong adhesion are obtained in the CC‐LTO@CNTs core/shell arrays. The electrochemical performance of the CC‐CNTs/LTO electrode is completely studied as the anode of LIBs and it shows noticeable high‐rate capability (a capacity of 169 mA h g^−1^ at 1 C and 112 mA h g^−1^ at 20 C), as well as a stable cycle life with a capacity retention of 86% after 5000 cycles at 10 C, which is much better than the CC‐LTO counterpart. Meanwhile, excellent cycling stability is also demonstrated for the full cell with LiFePO_4_ cathode and CC‐CNTs/LTO anode (87% capacity retention after 1500 cycles at 10 C). These positive features suggest their promising application in high‐power energy storage areas.

Over the past decade, power lithium‐ion batteries (LIBs) have developed rapidly to meet the growing market of hybrid electric vehicles and electric vehicles. The performance of the power LIBs is mainly controlled by the electrode materials.^1^, ^2^, ^3^, ^4^, ^5^ However, the commercial graphite anode cannot meet the power requirements due to compromised high‐rate performance arising from low ionic/electronic diffusion coefficient and oxidation issue at large working current densities.^6^, ^7^, ^8^, ^9^, ^10^ Therefore, great efforts are made to exploring advanced high‐power electrode materials.

Of anode candidates, spinel Li_4_Ti_5_O_12_ (LTO) has been widely studied as an ideal high‐power anode of LIBs for fast energy storage/release.^11^, ^12^, ^13^, ^14^ As a zero‐strain insertion material, LTO can maintain initial structure and have a fast phase transformation process at high‐rates.^15^, ^16^ Nevertheless, the LTO is a p‐type semiconductor with low electronic conductivity, which cannot support fast electron transfer required by high rates. Meanwhile, the ion transport path is too long in the bulk LTO electrode leading to slow reaction kinetics.^17^ To circumvent these issues, many strategies have been taken to modify LTO to achieve high performance. Typically, one effective route is to take nanoporous design on LTO to obtain various LTO nanostructures (nanoparticles,^18^ nanosheets,^19^ nanorods,^20^ etc.), which possess shortened transport channels for ions/electrons. Despite great progress, the single nanoporous design on LTO is not enough because the electron transfer rate is still limited in the LTO nanostructures. In such a context, another better route is to combine LTO nanostructures with conductive matrixes (such as carbon, metal, and conducting polymers).^21^, ^22^, ^23^ While metal and conducting polymer matrixes are prone to produce adverse side reactions at high working voltage leading to oxidization or degradation. Hence, to date, carbon matrix is considered as the most popular candidate to be fitted together with LTO.

Currently, there are several works on the LTO/carbon powder composites including LTO/ reduced graphene oxide (RGO),^23^ LTO/amorphous carbon,^21^ and LTO/carbon nanotubes (CNTs),^24^ and LTO/carbon fibers.^25^ Due to use of insulting binders and postpressing technique, the performance of the powder samples would be greatly undermined. In recent years, integrated arrays electrodes are becoming the research pet because of their great potential application in wearable equipment and microelectronics. Carbon cloth supported CNTs (CC‐CNTs) arrays emerge and show high electronic conductivity, excellent chemical stability, and mechanical flexibility.^26^, ^27^ Without additional binders, the integrated CNTs arrays in combination with other active materials could provide better conductive network for fast electron transfer and large surface area.^28^, ^29^, ^30^, ^31^, ^32^ As a result, it could improve the transfer kinetics of ions/electrons in the whole electrode to obtain enhanced electrochemical performance, especially at high‐rate application. As the saying goes: “a fence of the three piles, one of the three men to help.” Atomic layer deposition (ALD) is the right man for the CC‐CNTs arrays due to its reproducibility, simplicity, and high uniformity of the deposited materials.^33^ ALD can help to realize controllable synthesis of active materials on CC‐CNTs arrays with no damage on CNTs arrays. Therefore, it would be very interesting to explore the directional combination between CC‐CNTs arrays and ALD‐LTO, and now this research is still in blank filed.

Herein, we report a powerful strategy to achieve rational construction of CC‐CNTs/LTO core/shell arrays with the help of combined chemical vapor deposition (CVD)–ALD method. The CNTs arrays used here are directly grown on CC acting as current collector without additive binder and metal collector. The network made up of CNTs arrays provides porous conductive channels for electron transport and exhibits a large specific surface area which offers a large number of active sites for ion insertion/extraction. The ALD technique ensures uniform deposition of high‐quality LTO layers on the CC‐CNTs arrays. These positive advantages make the CC‐CNTs/LTO core/shell arrays exhibit remarkable high‐rate capability (a capacity of 169 mA h g^−1^ at 1 C and 112 mA h g^−1^ at 20 C) and cycling performance. Furthermore, the full cells with LiFePO_4_ cathode and CC‐CNTs/LTO anode are also assembled and demonstrated with outstanding cyclic stability with a capacity retention of 87% after 1500 cycles at 10 C. Our results demonstrate a novel high‐power anode and the usefulness of ALD for construction of advanced electrodes for application in energy storage and conversion.

The CC‐CNTs/LTO composite electrode is synthesized by the combined CVD–ALD lithiation method. As illustrated in **Figure** **1**a, first, random CNTs arrays are grown on CC substrate by CVD. Then, ALD‐TiO_2_ is uniformly coated on the CNTs' skeleton forming CNTs/TiO_2_ core/shell arrays. Finally, the CNTs/TiO_2_ arrays are converted into CNTs/LTO core/shell arrays by a facile chemical lithiation process. Note that the CC‐CNTs/LTO composite electrode is flexible and could be directly acted as electrode for LIBs. Moreover, according to the thermogravimetric analysis (TGA), the contents of LTO, CNTs, and CC in the whole electrode are 11.45%, 10.88%, and 77.67%, respectively (Figure S1, Supporting Information). The total carbon content in the electrode is 88.55%.

**Figure 1:**
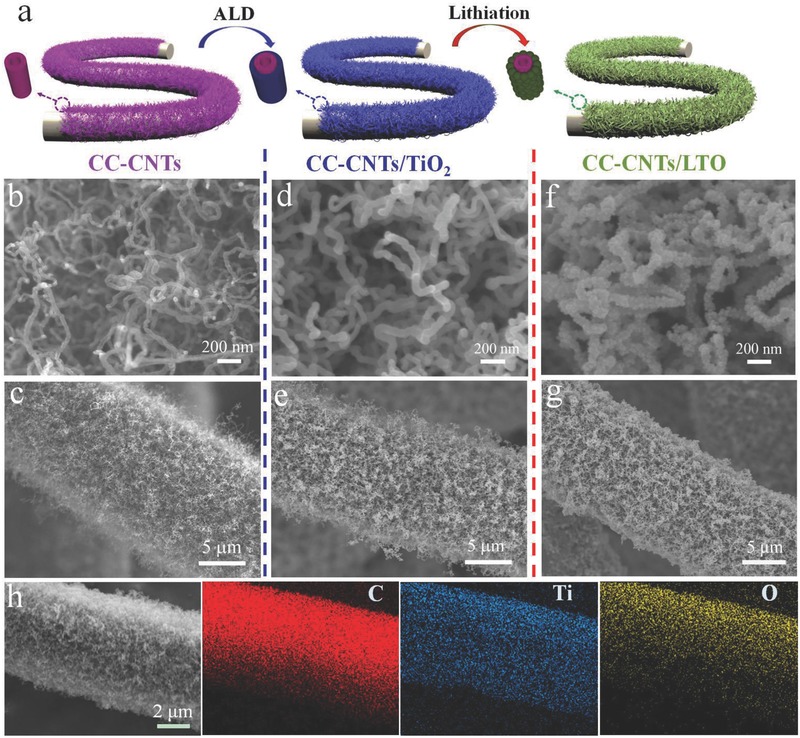
a) Fabrication schematics of CC‐CNTs/LTO core/shell arrays; SEM images of b,c) CC‐CNTs arrays, d,e) CC‐CNTs/TiO_2_ arrays, and f,g) CC‐CNTs/LTO arrays; h) EDX mapping images of C, Ti, and O elements in CC‐CNTs/LTO electrode.

The morphologies of products at different stages are examined by scanning electron microscopy (SEM). SEM images (Figure 1b,c and Figure S2a, Supporting Information) show that the CC substrate is homogeneously coated by interconnected CNTs with diameters of 20–50 nm forming a uniform porous network. Without any additional insulating binders and additives, random CNTs arrays directly grown on CC show good electronic conductivity and superior flexibility with promising application in flexible devices. Moreover, each CNT is in contact with the subtract which could facilitate fast electron transfer. After ALD process, the TiO_2_ layer of ≈10 nm is homogeneously deposited on the surface of each CNT and the whole 3D porous network structure is still well maintained (Figure 1d,e and Figure S2b, Supporting Information). Finally, after chemical lithiation process, the continuous TiO_2_ layer is converted into interconnected LTO nanoparticles of 15–20 nm, which is strongly anchored on the CNTs forming final porous CNT/LTO core/shell arrays (Figure 1f,g and Figure S2c, Supporting Information). The appearance of the CNTs/LTO core/shell structure becomes rougher and the diameter increases up to 70–80 nm. Impressively, the integral electrode keeps excellent flexibility and could be randomly bent and folded (Figure S2d, Supporting Information). In addition, the specific surface area of CC‐CNT/LTO core/shell arrays is tested by Nitrogen adsorption–desorption measurement. It shows a specific surface area of about 98 m^2^ g^−1^, which is favorable for sufficient contact between electrode and electrolyte (Figure S3, Supporting Information). Furthermore, the distribution of LTO nanoparticles on CNTs is also supported by energy dispersive X‐ray spectroscopy (EDX) mapping (Figure 1h). It is seen that Ti, O, and C elements are uniformly distributed on the CC, indicating the homogeneity of LTO.

Detailed microstructures of samples at different stages are further investigated by transmission electron microscopy (TEM)‐high‐resolution transmission electron microscopy (HRTEM). As shown in **Figure** **2**a,b, the hollow structure of CNTs is clearly seen with internal diameters of ≈10 nm and external diameters of ≈30 nm. The lattice spacing of 0.34 nm in HRTEM image (Figure 2b) is in consistent with the (002) interplanar distance of graphite carbon (JCPDS 75‐1621), corresponding to the selected‐area electron diffraction (SAED) pattern (inset image in Figure 2a). After ALD process, only the polycrystalline rings of CNTs could be seen in the SAED pattern (inset image in Figure 2c) and no obvious ring/lattice fringe of ALD‐TiO_2_ is detected (inset image in Figure 2d). It indicates the amorphous nature of ALD‐TiO_2_ layer. Moreover, the external diameter of nanotube of CC‐TiO_2_ increases to 50–60 nm (Figure 2c,d). It suggests that the thickness of outer TiO_2_ layer is ≈10 nm and the inner structure of CNTs is still well preserved. After chemical lithiation process, polycrystalline LTO nanoparticles are fully coated on the CNTs, forming CNTs/LTO core/shell composite with a diameter of 70–80 nm (Figure 2e,f). The corresponding SAED pattern (inset image in Figure 2e) exhibits three rings, in agreement with (111), (400), and (440) planes of the spinel lithium titanate phase (JCPDS 49‐0207). Furthermore, the HRTEM analysis of LTO (Figure 2f) reveals that the lattice spacing of 0.48 nm is in consistent with (111) plane of LTO.

**Figure 2:**
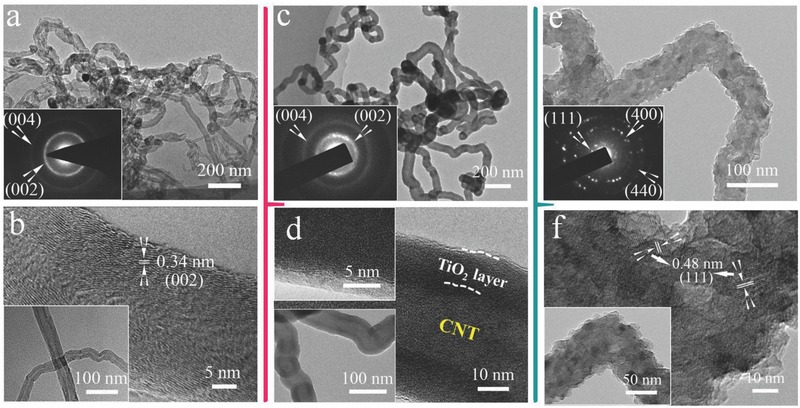
TEM and HRTEM images of a,b) CC‐CNTs arrays (SAED image in inset), c,d) CC‐CNTs/TiO_2_ core/shell structure (SAED image in inset), and e,f) CC‐CNTs/LTO core/shell structure (SAED images in inset).

The crystallographic structure and phase evolution of samples are also studied by X‐ray diffraction (XRD; **Figure** **3**a). For the CC‐CNTs/LTO electrode, diffraction peak at ≈26° is corresponding to the pristine CC and CNTs, in consistent with (002) plane of graphite (JCPDS 75‐1621). Except for the above diffraction peaks, the rest peaks belong to spinel LTO phase (JCPDS 49‐0207). In addition, the XRD pattern of CC‐CNTs/TiO_2_ electrode is similar to that of the CC‐CNTs electrode, suggesting the amorphous nature of ALD‐TiO_2_. And the existence of TiO_2_ is further characterized by Raman spectroscopy (Figure 3b). Apart from D band (1351 cm^−1^) and G band (1586 cm^−1^) of carbon, the Raman spectrum of CC‐TiO_2_ shows three broad peaks at 239, 403, and 608 cm^−1^, corresponding to the characteristic peaks of TiO_2_.^34^, ^35^ As for CC‐CNTs/LTO, three typical peaks of LTO located at 231, 424, and 679 cm^−1^ can be observed, in agreement with the vibration mode of Li−O bands (3F_2g_), vibrations of Li−O ionic bands located in LiO_4_ tetrahedral (E_g_), and the stretching vibrational mode of Ti−O bands in the TiO_6_ octahedral (A_1g_).^36^ Furthermore, the surface state of the CC‐CNTs/LTO composite is also characterized by X‐ray photoelectron spectroscopy (XPS). The characteristic peaks of O 1s, Ti 2p, C 1s, and Li 1s are detected in the survey spectrum (Figure 3c). The high‐resolution spectrum of Ti 2p (Figure 3d) shows two obvious peaks centered at 458.7 and 464.4 eV associated with the Ti 2p_1/2_ and Ti 2p_3/2_ state of Ti^4+^ and no other valence state of Ti is found. From the O 1s spectrum (Figure 3e), the peaks at around 530.1 and 531.5 eV could be indexed to a prominent peak of Ti—O bond (530.1 eV) and —OH bond (531.5 eV), respectively.^37^, ^38^ As for the C 1s spectrum (Figure 3f), it presents C—C bond (284.6 eV) and C—OH bond (286.2 eV). Therefore, from the above results, it is reasonable to verify the successful synthesis of CC‐CNTs/LTO composite electrode.

**Figure 3:**
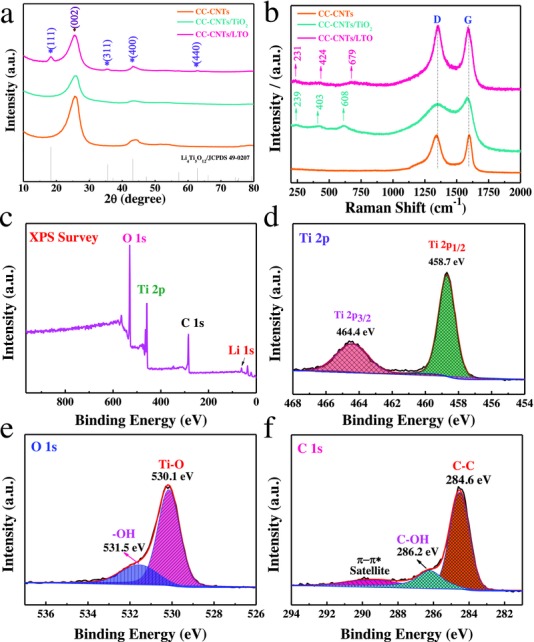
Phase and composition characterizations of CC‐CNTs, CC‐CNTs/TiO_2_, and CC‐CNTs/LTO arrays: a) XRD patterns; b) Raman spectra. XPS spectra of CC‐CNTs/LTO core/shell composites: c) survey, d) Ti 2p, e) O 1s, and f) C 1s.

The Li‐ion storage performance of the CC‐CNTs/LTO composite electrode is thoroughly characterized as anode of LIBs. For comparison, we also prepared LTO on CC (CC‐LTO; see Figures S2e,f and S4, Supporting Information) via the ALD‐lithiation method as the same parameters. **Figure** **4**a shows the comparison of CV curves at a scan rate of 1 mV s^−1^ at the 1st cycle in the voltage range from 2.5 to 1.0 V (vs Li/Li^+^). Comparatively, the CC‐CNTs electrode shows very little CV area and no active redox couple is noticed in the voltage range from 2.5 to 1.0 V. Moreover, the galvanostatic charge–discharge profiles of CC‐CNTs electrode exhibits a little capacitance of less than 8 mA h g^−1^ (Figure S5e, Supporting Information). All above indicates that the CC‐CNTs electrode mainly servers as the current collector and their capacity contribution is less than 3.5%. In contrast, both CC‐LTO and CC‐CNTs/LTO electrodes exhibit a strong pair of redox peak at around 1.55 V (vs Li/Li^+^), due to the phase conversion between Li_4_Ti_5_O_12_ and Li_7_Ti_5_O_12_.^39^ Note that the peak current densities (*I*_p_) of CC‐CNTs/LTO electrode are much stronger than those of CC‐LTO electrode, and accompanied by smaller voltage gap (|∆*E*_P_|) between the redox peaks (Table S1, Supporting Information) at different scan rates (Figure S5a,c, Supporting Information). It suggests that the CC‐CNTs/LTO has a higher electrochemical reactivity, smaller polarization, and lower electrode resistance. Moreover, the CV is often used to calculate the lithium‐ion diffusion coefficient *D*_Li_ (cm^2^ s^−1^) according to the following equation^40^, ^41^

(1) $$I_{p}=2.69\times 10^{5}ACD^{1/2}n^{3/2}V^{1/2}$$

where *I*_p_ and *A* represent the peak current and surface area of the electrode, respectively. *C* is the concentration of lithium ion, *n* is the number of electrons per molecule during oxidization, and *V* is the scan rate. The relation between the *I*_p_ and *V* is illustrated in Figure S5b,d (Supporting Information). The value of *D*_Li_ is calculated and listed in Table S1 (Supporting Information). Obviously, the CC‐CNTs/LTO electrode exhibits higher diffusion coefficient (3.5 × 10^−9^ vs 5.4 × 10^−9^) due to its large surface area and highly porous structure benefiting from CNT arrays.

**Figure 4:**
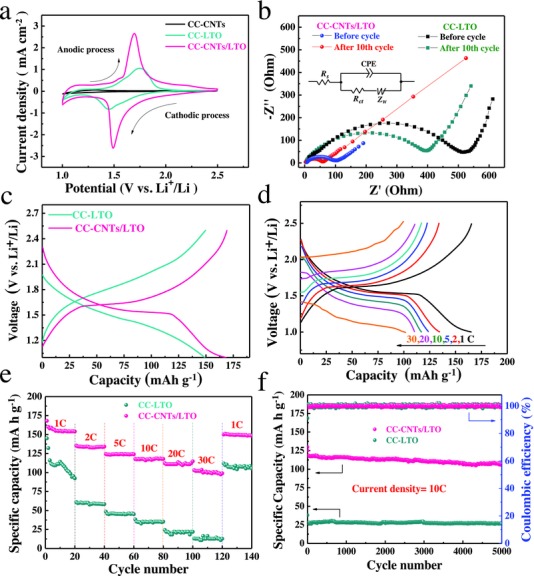
Electrochemical properties of CC‐CNTs/LTO core/shell electrodes for LIBs: a) CV curves at a scan rate of 1 mV s^−1^; b) Nyquist plots before cycle and after the 10th cycle. Inset: a simplified equivalent circuit. *R*_S_: ohmic resistance of solution and electrodes; *R*_ct_: charge‐transfer resistance; CPE: constant phase element; *Z*_W_: Warburg impedance. c) Comparison of charge/discharge curves at 1 C at the first cycle; d) galvanostatic charge–discharge profiles at different rates; e) high‐rate capability and f) cycling performance at 10 C.

To further study the ion/electron transport efficiency, electrochemical impedance spectroscopy (EIS) measurements are carried out and shown in Figure 4b. Both samples exhibit a characteristic Nyquist plot with a semicircle in high frequency regions owing to charge‐transfer resistance and a straight line in low frequency regions related to Warburg impedance of Li‐ion diffusion into the active material.^38^, ^42^, ^43^ The *R*_s_ (ohmic resistance) and *R*_ct_ (charge‐transfer resistance) of both samples after the 10th cycle are listed in Table S1 (Supporting Information). Whether before cycle or after the 10th cycle, it should be noted that the CC‐CNTs/LTO electrode performs lower *R*_s_ and *R*_ct_ than CC‐LTO counterpart. Both results from CV and EIS analysis demonstrate the better reaction kinetics of CC‐CNTs/LTO composite electrode due to the enhanced electronic conductivity arising from superior conductive network of CNTs arrays.

The charge/discharge curves of both samples at 1 C are shown in Figure 4c. The CC‐CNTs/LTO electrode exhibits lower charge plateau and higher discharge plateau, indicating smaller polarization and inner resistance. Moreover, the charge/discharge profiles of CC‐CNTs/LTO electrode at different current rates from 1 to 30 C are used to further study its high rate capability (Figure 4d). As the current rate increases, the charge/discharge profiles could remain flat plateaus. The specific capacities at every current rate are illustrated in Figure 4e and Figure S5f (Supporting Information). Obviously, the capacities of CC‐CNTs/LTO are higher than CC‐LTO at various current rates (169, 134, 125, 117, 112, 102 mA h g^−1^ vs 148, 59, 44, 35, 22, 12 mA h g^−1^ at 1, 2, 5, 10, 20, 30 C, respectively), especially at the high rate of 30 C (capacity retention 60% vs 8%). When it returns to 1 C, the capacity retention of the CC‐CNTs/LTO is 90%, higher than the CC‐LTO (75%). Furthermore, the designed CC‐CNTs/LTO electrode exhibits preeminent cycling stability at 10C (Figure 4f). A discharge capacity of 107 mA h g^−1^ is maintained after 5000 cycles and keeps 86% retention. On the contrary, the CC‐LTO electrode cannot undergo high‐rate cycling, and shows a very low specific capacity of 28 mA h g^−1^ at 10 C. In addition, except for the first Coulombic efficiency (91% vs 72%), the Coulombic efficiency of both samples is nearly maintained at 100%. Importantly, the CC‐CNTs/LTO electrode also exhibits much better performance than other counterparts (**Figure** **5**a) including graphene/LTO and sponge carbon/LTO composites in the literature.^44^, ^45^, ^46^, ^47^, ^48^ It is noted that our electrode shows excellent rate capability, especially at high rate, compared to other LTO‐based materials. Taking the current rate of 20 C, for example, the CC‐CNTs/LTO electrode exhibits a capacity of 112 mA h g^−1^, which is much higher than 73 and 25.7 mA h g^−1^ of LTO particles^46^ and similar LTO@CNTs composite,^48^ respectively. All above results demonstrate the ultrahigh‐rate performance of our electrode. In a nutshell, enhanced high‐rate performance has been demonstrated in the CC‐CNTs/LTO electrode, mainly due to the following beneficial factors. (1) Integrated porous conductive network from CNTs arrays can establish high transfer ways for ion/electrons and alleviate the external pressure during Li^+^ insertion/extraction due to the array architecture. (2) Large surface area of CNTs/LTO arrays provides sufficient contact between LTO and electrolyte and offers a large amount of reaction sites during electrochemical tests.^49^, ^50^, ^51^ (3) ALD‐synthesized LTO can be strongly anchored on the CNTs' skeleton to ensure stable high‐rate cycling. (4) The LTO nanoparticles have diameters of about 20 nm, which shortens the electron/ion transfer pathway and lowers the resistance. Therefore, both high Li^+^ diffusion efficiency and low resistance could ensure the fast reaction of Li_4_Ti_5_O_12_/Li_7_Ti_5_O_12_ at high rates, ensuring the excellent high‐rate capability of the electrode. (5) The free‐standing electrode could avoid the introduction of binder which will lower the conductivity and increase the inactive weight of the whole electrode. All these advantages above enhance the electronic/ionic conductivity of the entire electrode and lead to preeminent high‐rate and stable electrochemical performance.

**Figure 5:**
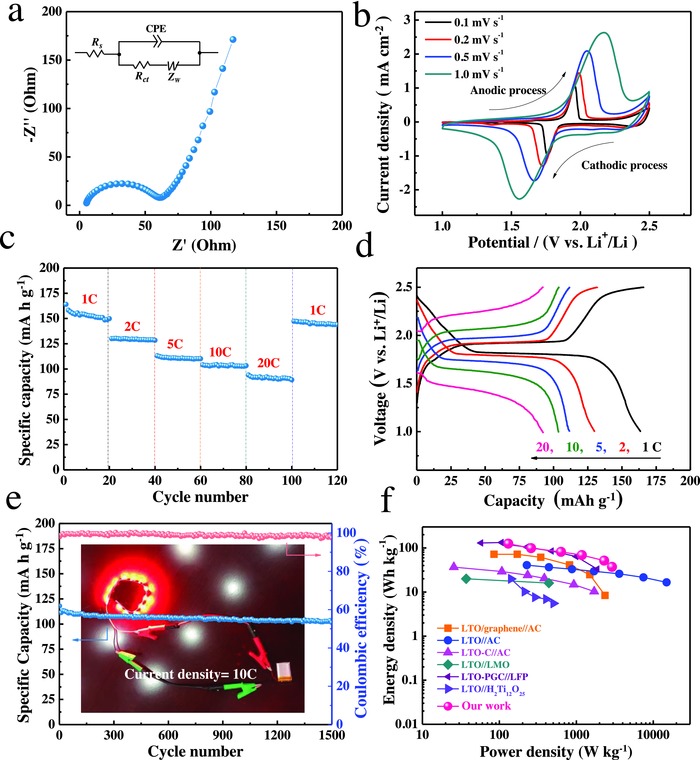
Electrochemical properties of LTO//LFP full battery: a) Nyquist plots after the 10th charge. Inset: a simplified equivalent circuit. *R*_S_: ohmic resistance of solution and electrodes; *R*_ct_: charge‐transfer resistance; CPE: constant phase element; *Z*_W_: Warburg impedance. b) CV curves at different scan rates; c) high‐rate capability; d) galvanostatic charge–discharge voltage profiles at different rates; and e) cycling performance and Coulombic efficiency at 10 C. Inset: photograph of 12 red LEDs powered by LTO//LFP full battery. f) Comparison of Ragone plot of energy and power densities of LTO‐based full batteries in the literature (LTO/graphene//AC,^41^ LTO//AC,^52^ LTO‐C//AC,^53^ LTO//LMO,^54^ LTO‐PGC/LFP,^55^ and LTO//H_2_Ti_12_O_25_ 56).

To demonstrate the application potential, we assembled full cell with the CC‐CNTs/LTO anode and commercial LiFePO_4_ (LFP) cathode. From the Nyquist plot (Figure 5a), the *R*_s_ and *R*_ct_ of the full battery are 4.5 and 60.4 Ω, respectively, which are a little larger than those of CC‐CNTs/LTO electrode, indicating that the inner resistance of the full cell is still small and would be beneficial for high‐rate application. The CV curves at different scan rates from 0.1 to 1.0 mV s^−1^ are shown in Figure 5b. Just one redox peak is noticed for all CV curves. Taking the CV curve at 0.1 mV s^−1^, for example, the full cell exhibits only a pair of redox peaks at about 1.85 V, matching well with the voltage difference between LiFePO_4_ (3.5 V vs Li/Li^+^) and Li_4_Ti_5_O_12_ (1.55 V vs Li/Li^+^). In our case, the full cell delivers a specific capacity of 164 mA h g^−1^ at 1 C, 130 mA h g^−1^ at 2 C, 111 mA h g^−1^ at 5 C, 103 mA h g^−1^ at 10 C, and 91 mA h g^−1^ at 20 C (Figure 5c). In addition, the corresponding galvanostatic charge/discharge profiles with a voltage platform of around 1.85 V, in agreement with the CV results (Figure 5b). It also presents stable cycling performance over 100 mA h g^−1^ at 10 C even after 1500 cycles, with a capacitance retention of 87% and Coulombic efficiency of nearly 100% (Figure 5e). In addition, to demonstrate the applications, 12 red light‐emitting diodes (LED) can be easily powered by LTO//LFP full battery (inset in Figure 5e). Moreover, we also compared the energy and power density of our full battery (based on LiFePO_4_ cathode and CC‐CNTs/LTO anode) with other LTO‐based full batteries (Figure 5f). Our LTO//LFP full battery could achieve a high energy density of 38 W h kg^−1^ at a high‐power density of 3 kW kg^−1^. The superiority of our work is evident. To sum up, all results above reveal the promising high‐rate application of the CC‐CNTs/LTO//LFP full battery.

In summary, we have proven the rational construction of CC‐CNTs/LTO core/shell arrays fabricated by a powerful combined CVD–ALD method. Novel integrated CC‐CNTs arrays are developed as the skeleton for the growth of ALD‐LTO. Combined properties of good flexibility, large surface area, and high conductivity are achieved in the CC‐CNTs/LTO core/shell arrays. Due to such interesting features, the CC‐CNTs/LTO core/shell arrays possess enhanced electrochemical kinetics, leading to excellent high‐rate capability and long cycle life for both half coin cells (86% capacity retention after 5000 cycles at 10 C) and full batteries of LTO//LFP (87% capacity retention after 1500 cycles at 10 C). Our work shows a new route to fabricate advanced flexible high‐power electrodes for applications in electrochemical energy storage.

## Experimental Section

*Materials Synthesis: Synthesis of CC‐CNTs/LTO Electrode*: First, CNTs arrays on CC were synthesized by a facile CVD with ethanol as the carbon precursor. The CC substrate was immersed in Ni(NO_3_)_2_ ethanol solution for 4 h and dried under 60 °C. Then the CC with catalyst was put into a tube furnace and treated at 600 °C under a mixed‐gas atmosphere of 140 sccm Ar + 10 sccm H_2_ for 30 min. Then Ar + H_2_ gas saturated with ethanol was introduced in the furnace for 90 min. Then, the sample was immersed into a solution with 1 m HCl and 1 m FeCl_3_ at 80 °C for 12 h to remove the Ni catalyst to form CC‐CNTs arrays. Second, TiO_2_ was fabricated on CNTs by an ALD (Beneq TFS 200) method using TiCl_4_ and H_2_O as the Ti and O precursors, respectively. Finally, the CC‐CNTs/TiO_2_ electrode was converted into CC‐CNTs/LTO by a simple chemical lithiation method. The as‐prepared CC‐CNTs/TiO_2_ electrode was placed in a 100 mL Teflon‐lined autoclave filled with 70 mL 3 m LiOH aqueous solution for 1 h at 80 °C and annealed under Ar atmosphere at 500 °C for 2 h to form CC‐CNTs/LTO core/shell arrays. The dependence of capacity and LTO mass was shown in Figure S6 (Supporting Information). The capacity would decrease as the LTO mass increases. Here, 2 mg cm^−2^ was selected as the representative.

*Materials Synthesis: Synthesis of CC‐LTO Electrode*: The CC‐LTO electrode was synthesized by the same ALD‐lithiation method described as above. To make the comparison more accurate, the load mass of LTO in the CC‐LTO electrode was also 2.0 mg cm^−2^.

*Materials Characterization*: The morphologies and microstructures of the as‐prepared samples were characterized by SEM (Hitachi S‐4800) and TEM (FEI Tecnai G2 F20 at 200 kV). XRD was performed on a Rigaku D/MAX 2550/PC X‐ray diffractometer to study the phase and crystal structures. Raman spectra were carried out with a LabRamHRUV Raman system under laser excitation at 514 nm. XPS was performed by an eSCALAB250Xi system. The Brunauer–Emmett–Teller (Autosorb‐1‐C) test was performed to calculate the specific surface area. TGA was tested on a TA Q600 (TE, USA) apparatus in air from room temperature to 900 °C at a heating rate of 5 °C min^−1^.

*Electrochemical Measurement*: Electrochemical measurement was carried out using two‐electrode coin cells (CR 2025), assembled with CC‐CNTs/LTO (working electrode), lithium foil or commercial LiFePO_4_ (counter electrode), and microporous polypropylene film (Celgard, 2300) as the separator in an Ar‐filled glove box. The mass ratio of LTO and LFP is 1:1.1. The electrolyte solution consisted of 1 m LiPF_6_ in ethylene carbonate/dimethyl carbonate (1:1, by volume). Cyclic voltammetry measurements were performed on a CHI 660E electrochemical workstation (CH Instruments Inc., Shanghai) from 1.0 to 2.5 V. The EIS results were tested by a Princeton Applied Research advanced electrochemical system over a frequency range of 100 kHz to 0.01 Hz. The galvanostatic charge/discharge studies were carried out using a LAND battery testing system between 1.0 and 2.5 V at room temperature. The capacity was calculated based on the mass of LTO.

## Conflict of Interest

The authors declare no conflict of interest.

## Supporting information

Supplementary
