# Supplementary material for: Smart Construction of Integrated CNTs/Li4Ti5O12 Core/Shell Arrays with Superior High‐Rate Performance for Application in Lithium‐Ion Batteries
Source: Adv Sci (Weinh). 2018 Jan 3;5(3):1700786. doi: 10.1002/advs.201700786 (PMC5867038; doi:10.1002/advs.201700786)
Supplement: Supplementary file 1 — Supplementary [file ADVS-5-1700786-s001.pdf]

Copyright WILEY-VCH Verlag GmbH & Co. KGaA, 69469 Weinheim, Germany, 2018.

ADVANCED SCIENCE Open Access

Supporting Information

for *Adv. Sci.,* DOI: 10.1002/advs.201700786

Smart Construction of Integrated CNTs/$Li_4Ti_5O_{12}$ Core/Shell Arrays with Superior High-Rate Performance for Application in Lithium-Ion Batteries

Zhujun Yao, Xinhui Xia, Cheng-ao Zhou, Yu Zhong, Yadong Wang, Shengjue Deng, Weiqi Wang, Xiuli Wang, and Jiangping Tu**

Supporting Information

Smart Construction of Integrated CNTs/$Li_4Ti_5O_{12}$ Core/Shell Arrays with Superior High-Rate Performance for Application in Lithium-Ion Batteries

By Zhujun Yao, Xinhui Xia,* Chengao Zhou, Yu Zhong, Yadong Wang, Shengjue Deng, Weiqi Wang, Xiuli Wang, Jiangping Tu*

Prof. X. H. Xia, Prof. J. P. Tu, Prof. X. L. Wang, Z. J. Yao, C. A. Zhou, Y. Zhong, S. J. Deng, W. Q. Wang

State Key Laboratory of Silicon Materials

Key Laboratory of Advanced Materials and Applications for Batteries of Zhejiang Province

School of Materials Science& Engineering

Zhejiang University, Hangzhou 310027, China

Email: helloxxh@zju.edu.cn; tujp@zju.edu.cn

Dr. Y. D. Wang

School of Engineering

Nanyang Polytechnic, 569830, Singapore

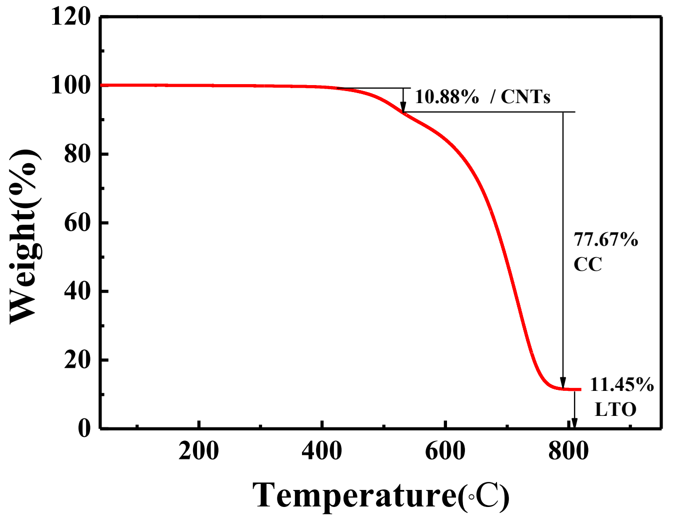

Figure S1. TGA profile of CC-CNTs/LTO electrode.

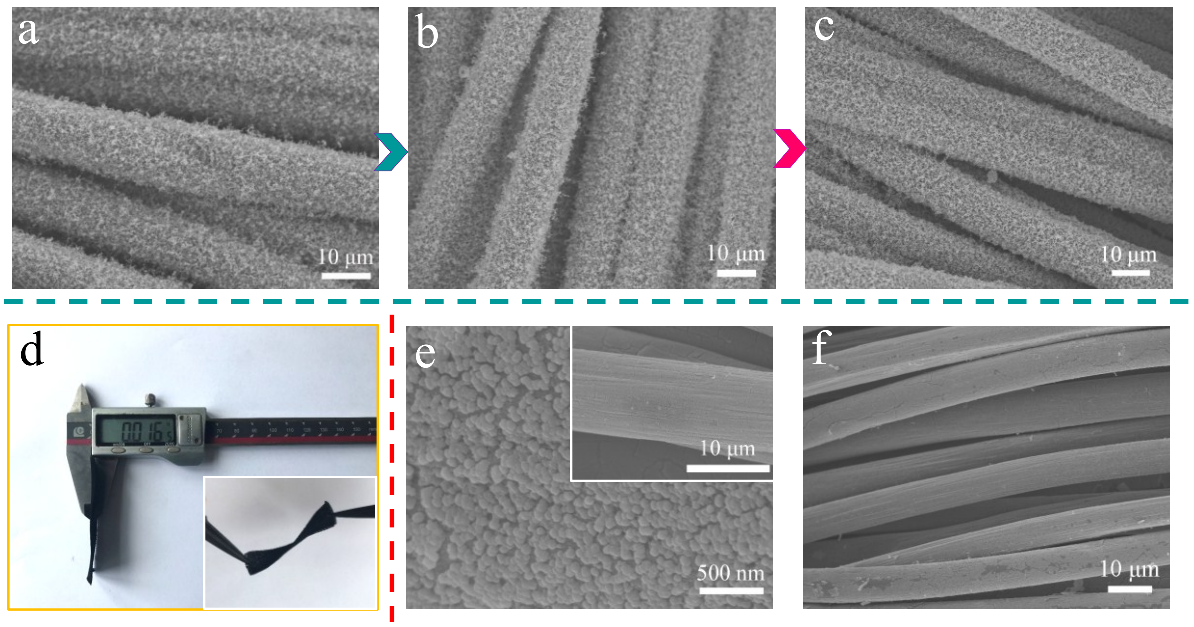

Figure S2. SEM images of (a) CC-CNTs arrays, (b) CC-CNTs/TiO_2 arrays and (c) CC-CNTs/LTO arrays; (d) Photo of the CC-CNTs/LTO core/shell electrode; (e-f) SEM images of CC-LTO (low magnification structure in inset).

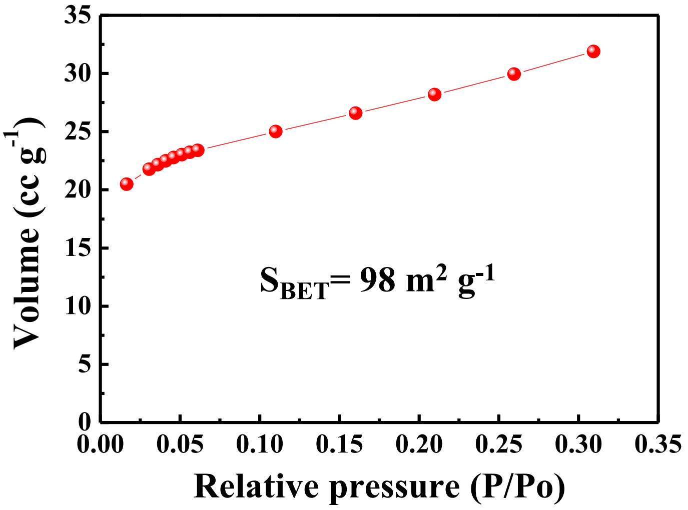

Figure S3. Nitrogen adsorption-desorption isotherm curve of the CC-CNTs/LTO arrays.

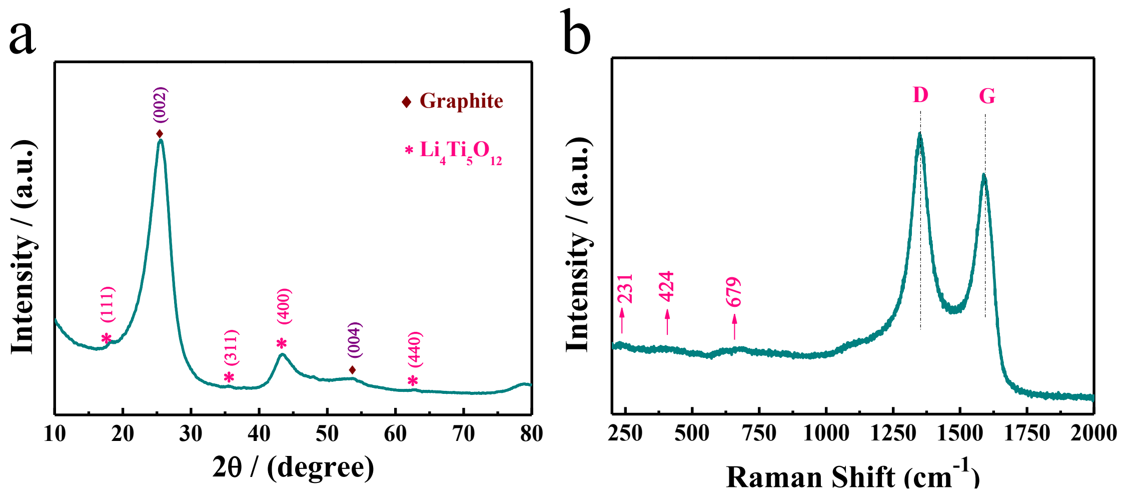

Figure S4. Phase and composition characterizations of CC-LTO composite: (a) XRD pattern and (b) Raman spectrum.

Discussion on SEM, XRD and Raman of CC/LTO composite (Figure S2e-f, Figure S4)

Figure S2e-f shows SEM images of CC/LTO composite. It is seen that the carbon cloth is uniformly covered with LTO nanoparticles, with a diameter of ca. 30-50 nm. The XRD pattern of the CC/LTO is shown in Figure S4a. The diffraction peaks at about 26° and 54° correspond to the (002) and (004) lattice planes of graphitic carbon (JCPDS 75-1621), respectively. Except for the peaks of carbon cloth, the other diffraction peaks can be indexed well with the spinel LTO phase (JCPDS 49-0207). In addition, Raman spectrum is shown in Figure S4b. Two characteristic peaks at ~1351 cm^{-1} (D band) and 1586 cm^{-1} (G band) are identified for the carbon cloth. Moreover, the peaks at ~231 cm^{-1} ($3F_{2g}$), 424 cm^{-1} (E_g) and 679 cm^{-1} (A_{1g}) are indexed to LTO, in consistent with the results of CC/CNTs/LTO. All above analysis identifies the success synthesis of CC/LTO.

Table S1 CV and EIS results of CC-CNTs/LTO and CC-CNTs electrodes

**Sample**	**I_p / mA cm^{-2}**	**$\|\Delta E_P\|$ / V**	**D_{Li} / cm^2 s^{-1}**	**R_s / Ω (before 1st/after 10th cycle)**	**R_{ct} / Ω (before 1st/after 10th cycle)**
**CC-CNTs/LTO**	2.65	0.21	3.5×10^{-9}	4.1/2.1	99.9/53.1
**CC-LTO**	1.07	0.31	5.4×10^{-10}	10.2/2.2	498.3/363.5

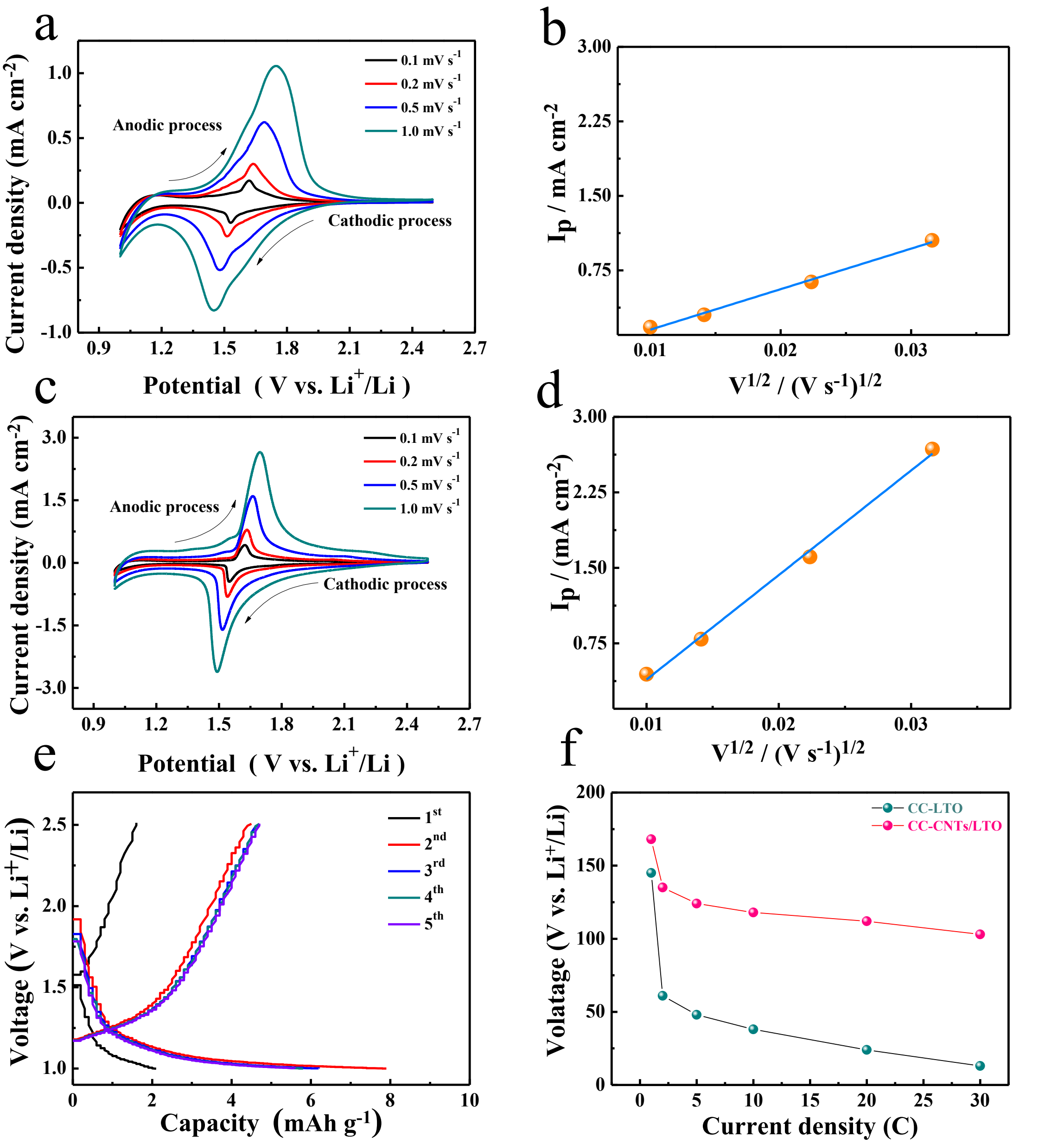

Figure S5. (a) CV curves at different scan rates and (b) Peak current I_p as a function of square root of scan rate $v^{1/2}$ of CC-LTO; (c) CV curves at different scan rates and (d) Peak current I_p as a function of square root of scan rate $v^{1/2}$ of CC-CNTs/LTO electrodes; (e) Galvanostatic charge-discharge voltage profiles of CC-CNTs at 175 mA g^{-1}; (f) Specific capacities (at different current rates) of CC-LTO and CC-CNTs/LTO electrodes.

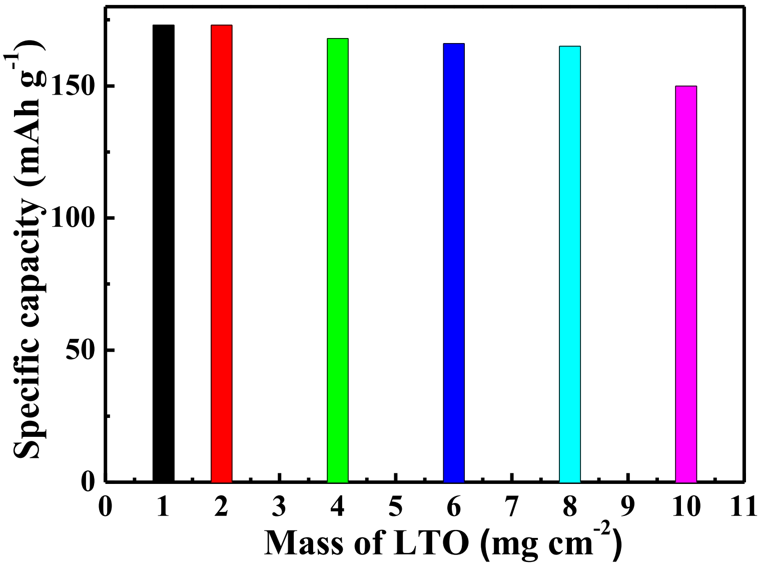

Figure S6. The dependence of capacity and LTO mass (at 1C).

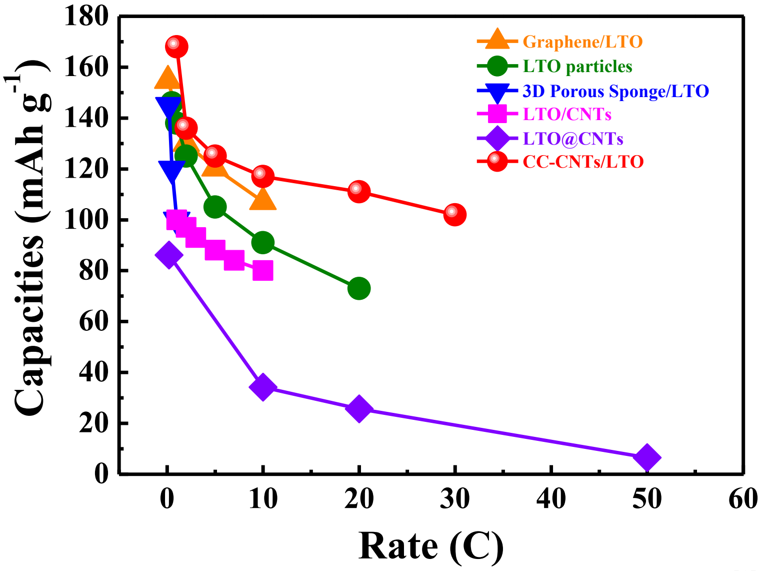

Figure S7. Comparison of rate capability in the literature (Graphene/LTO,[1]LTO particles,[2] 3D Porous Sponge/LTO,[3] LTO/CNTs,[4] LTO@CNTs[5]).

Reference

[1] J. Jiang, P. Nie, B. Ding, W. Wu, Z. Chang, Y. Wu, H. Dou, X. Zhang, *ACS Appl. Mater. Inter.* **2016**, *8*, 30926.

[2] J. Liu, Y. Shen, L. Chen, Y. Wang, Y. Xia, *Electrochim. Acta.* **2015**, *156*, 38.

[3] W. Liu, Z. Chen, G. Zhou, Y. Sun, H. R. Lee, C. Liu, H. Yao, Z. Bao, Y. Cui, *Adv. Mater.* **2016**, *28*, 3578.

[4] L. Deng, W.-H. Yang, X. Lyu, S.-F. Wei, Z. Wang, H.-X. Wang, *Chin. Chem. Lett.* **2017**, *28*, 362.

[5] J. Shu, L. Hou, R. Ma, M. Shui, L. Shao, D. Wang, Y. Ren, W. Zheng, *Rsc Adv.* **2012**, *2*, 10306.
